# Strengthening Health Workforce in Georgia: Identifying Gaps and Integrating Evidence‐Based Strategic Planning

**DOI:** 10.1002/hpm.3922

**Published:** 2025-03-30

**Authors:** Giorgi Aladashvili, Mariam Kirvalidze, Aleksandre Tskitishvili, Nikoloz Chelidze, Nikoloz Tvildiani, Giorgi Pkhakadze, Thomas J. Bossert, Karsten Lunze, Ilia Nadareishvili

**Affiliations:** ^1^ David Tvildiani Medical University Tbilisi Georgia; ^2^ Department of Neurobiology Aging Research Center Care Sciences and Society Karolinska Institutet and Stockholm University Stockholm Sweden; ^3^ Department of Global Health and Population Harvard T.H. Chan School of Public Health Boston Massachusetts USA; ^4^ Chobanian and Avedisian School of Medicine Boston University Boston Massachusetts USA; ^5^ Section of General Internal Medicine Department of Medicine Boston Medical Center Boston Massachusetts USA

**Keywords:** Georgia (Eastern Europe), health policy, health workforce planning, strategic planning, workforce sustainability

## Abstract

Health workforce planning is essential for ensuring a resilient and well‐functioning healthcare system capable of addressing population needs and responding to crises. In Georgia, an upper‐middle‐income country, significant challenges remain in the strategic planning, regulation, and management of the health workforce. This policy analysis evaluated health workforce planning approaches in Georgia's dynamic health system context. Health workforce planning in Georgia, guided by the National Health Strategy 2022–2030, prioritises needs‐based workforce planning, professional qualifications, and nursing development. However, Georgia faces data inconsistencies, workforce imbalances, and an uneven geographic distribution of healthcare professionals, limiting the efficacy of current policies. The lack of formal health workforce planning, reliance on market‐driven approaches, and weak retention strategies contribute to workforce shortages and migration. A centralised planning body, and enhancement in data collection and management, could facilitate the gradual introduction of context‐relevant, evidence‐based workforce planning methods. By integrating rigorous, long‐term workforce planning with intersectoral collaboration and adopting innovative methods like workload‐based modelling and hybrid planning methods, Georgia can create a sustainable health workforce aligned with its health system's evolving needs.

**Summary**

- Georgia's health workforce planning is framed in the National Health Strategy 2022–2030, but significant challenges (e.g., data inconsistencies and geographic imbalances) persist in the young post‐Soviet country (characterised by highly privatised health and education systems, insufficient professional self‐regulation).
- The current workforce is inadequately distributed across regions and specialities, with an overreliance on market‐driven approaches.
- A centralised planning body is recommended to lead strategic workforce planning efforts, improve data collection and management, and use scenario‐based modelling and hybrid workforce planning methods to ensure long‐term workforce sustainability.
- Strengthening professional development, particularly in nursing, and addressing migration are critical to improving workforce performance, retention and workforce balance.
- Intersectoral planning involving collaboration between health, education, and labour sectors is key to addressing long‐term workforce challenges, ensuring a more resilient healthcare system.

## 1 Introduction

A well‐functioning health workforce is fundamental for ensuring that health systems effectively meet population needs, maintain health service quality and accessibility, and provide systemic resilience during public health crises [1, 2, 3, 4]. Countries in the World Health Organisation (WHO) European Region currently face significant health workforce challenges, including inadequate strategic planning, deficient information systems, uneven geographic availability, poor governance, and substandard working conditions [3, 5, 6, 7, 8].

Effective health workforce planning aims to ensure that the right number of people with relevant skills are available at the right place and time [7]. National‐level planning helps countries forecast future health system needs by profession, speciality, location, and sector, allowing for proactive preparation. It guides the production of new professionals, retention, and optimisation of the existing workforce. Health workforce strategic planning is a technical process to predict demands for care and the staff required to provide it in the long‐term (usually three to 10 years, compared to operational planning, which usually spans less than a year). This approach has become widely adopted worldwide to address workforce challenges, with countries implementing it to varying levels of success [9, 10, 11, 12]. The lack of strategic planning has contributed to a health workforce crisis for European health systems, driven by labour shortages, mental health concerns, education gaps, gender disparities, and insufficient financial investment [5, 6, 13]. However, this crisis also presents an opportunity to rebuild and strengthen the workforce, fostering more resilient health systems, capable of providing sufficient services even when crises change demands.

Various health workforce planning approaches exist, such as demand‐based, supply‐based, and needs‐based approaches, often utilising scenario generation and modelling frameworks [12, 14, 15, 16, 17]. Demand‐based approaches typically consider factors like population changes, workforce density, insurance systems, health industry growth, working hours, bed occupancy rates, health literacy, quality of life, and labour market conditions. Supply‐based strategies focus on student enrolment, study duration, graduate output, employment opportunities, career paths, workforce migration, sectoral transitions, and licencing. It also accounts for workload, workplace dynamics, salaries, and demographics. Need‐based approaches, increasingly popular, use modelling and forecasting of population health and epidemiological data to plan for appropriate skill mix and productivity [12, 14]. The WHO's Workload Indicators of Staffing Need (WISN) tool, widely used in low‐ and middle‐income countries, utilizes service data and workers' available time to manage health and administrative tasks, promoting multi‐stakeholder engagement and governance [18]. We summarise several common health workforce planning methods in the Supporting Information S1: Table 1. Application of the planning methods and approaches varies among countries, including a high variability in Europe, usually driven by local contexts, priorities, and analytic planning capacities [19].

Georgia, an upper‐middle‐income country in Eastern Europe's South Caucasus with a population of 3.7 million, has consistently faced health worker challenges over the past years. Since Georgia's independence from the Soviet Union, there was minimal formal planning for human resources for health, so that appropriate supply and demand are unclear for physicians, nurses, nurse assistants, midwives, dentists, public health specialists, health managers, paramedics, and other professionals. In Georgia, the supply and demand of health professionals has depended largely on the labour market with little to no government involvement, making Georgia an example of ‘serendipitous replacement’ planning [6, 16]. To inform the health workforce planning and strengthening in Georgia, we evaluated evidence‐based health workforce planning approaches in the context of Georgia's health system and analysed the existing policies and plans. We propose actions for gradually introducing evidence‐informed planning in the country, complementing the basic outline in the National Health Strategy 2022‐2030 (NHS 2022‐2030).

## 2 Human Resources for Health in Georgia

### 2.1 Regulatory and Practice Environment

The Law of Georgia on Health Care and the Law on Medical Practice serve as the main frameworks governing medical practice, outlining licencing, responsibilities, rights, and governance [20, 21]. The Ministry of Internally Displaced Persons from the Occupied Territories, Labour, Health and Social Affairs of Georgia (referred to as ‘Ministry of Health’), and the Regulation Agency for Medical and Pharmaceutical Activities (referred to as ‘Regulatory Agency’), oversee and regulate medical practice and licencing in the country.

Georgia's healthcare system is largely privatised, with over 80% of hospital beds managed by for‐profit entities, the health workers' primary employers. The state operates rural primary care centres and a small number of public hospitals. Several international organisations support quality assurance, workforce training, and accreditation of these facilities. Six organisations are listed for medical facility accreditation, though facilities can also use unlisted bodies if they meet state requirements [22]. Accreditation requirements and standards vary but typically cover work processes, human resource management, and workforce development. However, varying accreditation standards and costs, pose a risk that most hospitals in Georgia opt for agencies with less stringent requirements and lower fees, potentially compromising healthcare quality. Ambulatory and laboratory facilities must comply with international standards organisation's (ISO) standards (ISO9001 for clinics and ISO15189 for laboratories) [22].

Professional associations and unions in Georgia are numerous. They play limited roles due to resource constraints, but they contribute to clinical guidelines, continuing medical education (CME), and professional development [23]. In 2023, the government announced formal recognition and registration of these associations for collaboration on healthcare system improvements [24].

### 2.2 Workforce Compensation and Wage Disparities

In 2021, the median monthly salary for physicians was 1500 GEL (∼550 USD), higher than the national median of 900 GEL. However, nurses and health and social service workers earned significantly less, with a median of 774 GEL (∼280 USD) [25]. In 2022, the government introduced a minimum hourly wage of 4.4 GEL (∼1.70 USD) for hospital nurses and 7 GEL (∼2.70 USD) for physicians, expanding in 2024 to include junior physicians, midwives, nurse assistants, and sanitary personnel. Although wages remain low, they are expected to gradually increase [22, 26]. Additionally, a significant wage disparity exists within the healthcare sector, with a minority of health workers earning 5 to 10 times the median salary, contributing to recognized inequality. Approximately 23% of physicians earn less than 500 GEL (∼180 USD) per month, while 14% earn over 5500 GEL (∼2000 USD) [23].

### 2.3 Physicians

Health workforce numbers reported by different organisations and documents vary, making effective planning difficult. For example, in 2021, the State Audit Office of Georgia indicated that the registry of certified physicians included 17,632 certified physicians, while in 2022, various government sources cited 22,490 to 24,000 [25, 27, 28]. The WHO dashboard listed 20,311 physicians in 2021, down from 28,291 in 2019, likely due to changes in data collection and reporting [29]. A notable discrepancy includes the sharp drop in the number of generalists, from 6772 in 2019 to just 1085 in 2020.

Physicians are unevenly distributed across Georgia, with Tbilisi, home to a third of the population, accounting for two‐thirds of all physicians [25, 30]. Tbilisi had a physician density of 905 per 100,000 people, compared to 395 per 100,000 in Imereti, the region with the second‐highest physician density [25]. Despite government programmes offering incentives for physicians to work in understaffed specialities and in Georgia's remote mountainous and border regions, such initiatives have had limited success, with a large portion of the budget unused and only 1266 rural physicians reported in 2022 [5, 30]. The reasons for the programme's limited success are unclear, but contributing factors may include low wages, lack of professional development opportunities, and lower living standards for physicians' families. Insufficient monitoring and weak political will to decentralise health services also play a role [31]. Additionally, rural areas face poor infrastructure like inadequate hygiene facilities, water supply, heating, and internet access [32, 33]. Some of these challenges are being addressed through government and international collaboration programmes, such as those with the WHO [34]. Imbalances in speciality numbers and an ageing primary care workforce further exacerbate health workforce challenges [5, 29].

To practice as a physician in Georgia, one must obtain an MD degree, pass the Unified Postgraduate Qualification Examination (multiple‐choice question‐based), complete a residency, pass the State Certification Exam (also multiple‐choice question‐based), and obtain the relevant speciality certification. Physicians can only practice independently in their certified specialities, except during emergencies. As of 2023, there are 27 specialities, some with sub‐specialities [35]. While certification is currently not time‐bound, the National Health Strategy 2022–2030 planned to introduce a re‐certification process by 2024 [36].

As of July 2024, the World Directory of Medical Schools listed 25 medical schools in Georgia, including 6 public and 19 private institutions. The National Centre for Educational Quality Enhancement (NCEQE) accredits 6‐year MD programmes, with World Federation for Medical Education (WFME) recognition valid until 2028 [37, 38]. The National Health Strategy 2022–2030 highlights the overproduction of MDs as a challenge [36].

**FIGURE 1:**
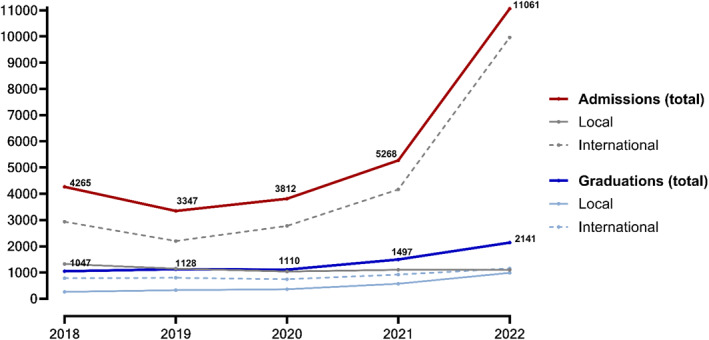
Annual MD degree admissions and graduations (ministry of education and science data).

Local applicants are admitted to MD programmes via the Unified National Examination, while international admissions vary by school. Local admission numbers have stabilised, with about 1000 MD graduates expected to join the labour market annually for the coming years. In contrast, international admissions more than doubled in 2022, reaching 9,964, partly due to students transferring from Ukraine (Figure 1) [34, 39]. Undergraduate medical education has become a major economic sector, contributing to foreign currency inflows [40].

In Georgia, specialised medical training is conducted through residency programmes lasting at least 3 years, depending on the speciality. The Regulatory Agency for Medical and Pharmaceutical Activities, under the Ministry of Health, oversees quality assurance and accreditation of residency programmes, but significant flaws exist [25]. A major issue is the lack of periodic self‐evaluation reports, essential for accreditation compliance. Programmes failing to submit these reports are not reassessed as required, undermining oversight. The NHS 2022–2030 aims to improve residency admissions and ensure all residency programmes meet World Federation for Medical Education standards by 2030. It also seeks to develop speciality competencies aligned with European criteria through collaboration with professional associations [36].

Residency candidates take the Unified Postgraduate Qualification Examination, a competitive MCQ‐based test [41, 42]. However, 75%–80% of the exam questions were publicly available as of 2024, many of which were outdated, misaligned with current curricula, and of poor quality [25]. Resident physicians are unpaid and must cover varying residency fees for training [38]. While the government funded priority specialities and offered support for those applying to train in high‐mountain and border areas for 3 years, by 2021, these initiatives covered less than 1% of total residency fees and had minimal impact [25].

Since 2008, Georgian physicians have received lifetime licences, and the mandatory Continuing Medical Education/Continuing Professional Development (CME/CPD) system was abolished due to corruption and mismanagement. CME is now voluntary for most specialities, with no robust quality assurance or updated courses. Even in specialities where CME is mandatory, many physicians fail to meet the 30‐credit minimum, with poor monitoring and low demand [25]. Stakeholders fear that reintroducing mandatory CME without quality control could make it a mere formality [23].

CME/CPD events can be accredited by the Ministry of Health, and international events can earn European CME credits through European Accreditation Council for Continuing Medical Education (EACCME), recognised by the Ministry upon request [38]. However, not all accredited events are held regularly, and most CME opportunities are concentrated in the capital, limiting access for physicians in other regions. Barriers to quality CME/CPD include financial constraints, lack of a CME/CPD culture, limited information, heavy workloads, and a shortage of relevant courses [23]. CME for nursing is even more challenging, but gradual CME requirements are expected with ongoing reforms. The NHS 2022–2030 envisions collaboration between the state and professional associations to develop CME programmes. Until CME is fully mandated, the government plans to incentivise CME participation through selective hospital contracting in state‐funded programmes [36].

### 2.4 Nurses

In 2022, government sources reported over 22,000 nurses (595.3 per 100,000 population), consistent with 2021 WHO data of 21,700, all female [27]. Between 1990 and 2019, physician density grew by 3.3% annually, while nurse density increased by only 1.6% [2]. The nurse‐to‐physician ratio declined from 2:1 to 1.1:1 (or 0.9:1 per the National Health Strategy) with an even lower ratio of 0.3:1 in primary care [2, 34, 36, 43].

In 2021, Georgia's nurse density was lower than in most high‐income European countries but comparable to Italy and Spain and higher than Israel and Greece [29]. With 37.5% of nurses over age 50, many are nearing or past retirement age of 60 [25]. Nurse density in Tbilisi (828 per 100,000) is higher than in most regions but not as high as for physicians [25]. Nursing faces challenges like low income, gender bias, and migration to the EU and the U.S., affecting workforce optimisation [44, 45, 46, 47]. The issue is not a severe numeric shortage, but challenges in nurse demographics, skills, and capacity building.

Nursing education in Georgia follows two paths: a 4 year bachelor's degree or a 3 year vocational training, with the latter producing most nurses. In 2022, 29 vocational colleges offered 1141 nursing positions, whereas only 19 students enrolled in bachelor's programmes, including 10 in English‐language courses. By 2023, only 60 undergraduate positions were available across three accredited bachelor's programmes.

Although the Nursing Development Strategy 2020–2030 prioritises bachelor's degrees, nursing remains unpopular, with only about 100 active bachelor's students in 2019. As a result, fewer than 20 nurses graduated annually with bachelor's degree. In contrast, most new nurses came from vocational programmes, with 493 graduates during the same period.

Nursing in Georgia was planned to become a regulated (certified/licenced) profession in 2025. Currently, nurses can work based on the education requirement, regardless of education being vocational, bachelor's or master's level. Under the new system, nurses will be classified as "certified" with a vocational degree, "registered" with a bachelor's, and "nurse practitioners" with a master's degrees. New graduates will be required to pass certification exams, while current practitioners will be registered without exams but will need to recertify every 5 years.

### 2.5 Other Professions

Pharmacist workforce data in Georgia is inconsistent, with fewer than 400 pharmacists reported annually from 2013 to 2023, despite pharmacy programmes enrolling more than 400 students each year [29]. In 2022, 22 vocational colleges offered 996 pharmacy positions, while 188 students enrolled in the Georgian‐language bachelor's programmes. In 2021, 400 students graduated from vocational pharmacy programs, with another 63 qualifying as pharmacy assistants. Like nursing, pharmacy will transition into a regulated profession under the NHS 2022‐2030.

The number of dentists in Georgia has more than doubled, rising from approximately 1000 in 2009 to 2464 in 2020—a 2.5‐fold increase [28]. In 2023, 13 accredited Doctor of Dental Medicine programmes offered 667 undergraduate seats through the Unified National Examination, excluding international students. In 2021, 47 dental technicians graduated from vocational colleges. Like nursing and pharmacy, according to the NHS 2022–2030, dentistry will become regulated professions.

As of 2022, Georgia had seven schools of public health offering master's programmes, three institutions providing PhD programmes, and one school with a bachelor's degree programme, the highest per capita in the former Soviet Union [40]. However, no workforce data is available for public health, epidemiology, physiotherapy, or other allied specialities.

## 3 Health Workforce Planning in Georgia

The primary document guiding health workforce development in Georgia is the National Health Strategy of Georgia 2022–2030 (NHS 2022–2030) [36]. It focuses on three pillars: needs‐based health workforce planning, enhancing professional qualifications, and the importance of nursing in the healthcare system. The document highlights the critical need for a highly qualified, geographically balanced, and accessible health workforce. The strategy primarily focuses on physicians and nurses while excluding dentists, pharmacists, and other healthcare or allied health professions.

The NHS 2022–2030 outlines key objectives such as improving needs‐based planning and aligning workforce production with regional and speciality healthcare demands. While specialities addressing the major burden of disease like surgery, cardiology, and oncology are likely to remain priorities, the strategy emphasises developing underrepresented fields like palliative care, geriatric medicine, and rehabilitation. It further stresses improving postgraduate medical education, including residency and continuing medical education (CME), to ensure professionals can meet evolving needs.

As the NHS 2022–2030 indicates a sufficient (or high) number of physicians per capita based on benchmarking, the focus shifts to increasing the nurse‐to‐physician ratio by raising the number of nursing programme graduates by 30% by 2025. Planned initiatives include establishing a licencing and certification system for nurses and ensuring 50% of them undergo continuing professional development. These efforts aim to elevate the nursing profession, improve job satisfaction, and enhance patient care–goals that align with Georgia's Nursing Development Strategy, adopted in 2019.

These strategic plans do not specify a methodology. Our assessment indicates insufficient baseline data, while projections were largely based on international benchmarks and expert opinions. This limits the documents' ability to address current and future population health needs [18]. Some planned actions in the strategies might be also limited in their power to bringing solutions. Defining workforce needs by speciality through consultations with relevant professional associations/unions, for example, can be constrained by the limited capacity and representativeness of professional associations [25]. There are limitations regarding workforce training: the strategy prioritises training in certain specialities and sub‐specialities without strong justification relative to other specialities affected by insufficient training. It also prioritises the wider introduction of the Objective Structured Clinical Examination (OSCE) in undergraduate medical education as a gold standard. However, the OSCE has its limitations and should not be seen as a comprehensive solution, particularly considering the integrity and professionalism of the educators and examiners. The extremely high number of both local and international MD students relative to the available bedside training opportunities in the country presents a major challenge but has received relatively little attention.

The strategy sets the objective of achieving compliance with WFME standards for postgraduate education. However, quality assurance in residency training is an ongoing international discussion, which involves the WFME and other stakeholders. These are just a few examples of areas in health workforce strategic planning that require urgent attention, including original research at the national level (such as medical and epidemiological studies) to inform the updating and development of clinical practice guidelines and standards. The country would benefit from a completely new and dedicated strategy, particularly considering that the implementation of the NHS 2022–2030 actions is already behind the set deadlines.

## 4 Roadmap for Strategic Planning in Georgia

### 4.1 Stewardship

Health workforce planning in Georgia should follow a stepwise approach, beginning with the establishment of a dedicated planning body staffed by ‘strategic HR thinkers’ [48]. This aligns with WHO recommendations to establish a health workforce planning and governance unit [13]. In the Framework for action on the health and care workforce in the WHO European Region 2023–2030, one of the indicators is a health workforce planning unit for the respective member state. Considering the efforts required to establish information systems, planning, and implementation within local and international health contexts, adequate investment is needed to equip a state‐funded unit with skilled management, research, and technical staff. A centralised planning body would be most effective in Georgia's current system. Governance should be flexible and adaptable to crises, with coordination mechanisms sustained and inclusive of stakeholder interests [49]. For successful participation, stakeholders must view the body as credible and see clear benefits from their involvement [48]. This body could be of a public‐private partnership format, bringing together the government officials, health workforce experts, employers, educators, international partners, and other stakeholders. Many European countries, including the Balkan nations of Romania and Bulgaria, Poland and the Baltic states, have units and offices under health ministries, overseeing planning activities [19, 50]. Planning environments and approaches vary a lot, however the experiences should be useful to consider in Georgia [19].

### 4.2 Terminology and Data

The planning process should proceed by introducing consistent terminology and definitions, such as ensuring clear distinctions between active workers, licenced professionals, and degree holders without licences. Developing robust data systems must be a priority for Georgia. Fragmented data collection and unreliable reporting hinder effective workforce planning, which depends on accurate, up‐to‐date baseline situational information [7]. Reliable and comprehensive baseline data is crucial for guiding planning, modelling, and tracking changes across key indicators. The NHS 2022–2030 acknowledged these data challenges and aimed to build a workforce registry for systematic data collection. To ensure its value, the data must be responsibly submitted, securely stored, and managed, requiring long‐term government commitment and oversight [49]. Workforce production and training data must be systematically reported and monitored. Even with limited resources, planning is possible using incomplete or outdated data, though it relies on certain assumptions [17, 51]. Qualitative analyses can help validate and contextualise assumptions and provide a clearer picture of the health system's dynamics [7, 12].

Personnel data should include the number of professionals by speciality, demographics, geographic distribution, attrition rates (migration, retirement, change in personal activity, leave, death), as well as overall workforce density. The planning process must also be guided by reliable data on services provided, required skill‐mix, available working time (for both health‐related and administrative tasks), workforce performance, motivation, migration intentions, job satisfaction, burnout, and professional development needs. The process requires accurate demand estimates, modelling, and consideration of integrated, interprofessional, and efficient patient‐ and population‐focused care, while also addressing the needs of patients and employers.

### 4.3 Integrating Evidence‐Based Health Workforce Planning in Georgia

Transitioning from simple benchmarking to more sophisticated workforce planning approaches, such as needs‐based, supply‐based, or demand‐based models, or their combinations (supply/demand projections) offers Georgia a pathway to develop an operational strategy that aligns workforce needs with health system demands. However, their long‐term planning must account for system dynamics, particularly in a context like Georgia, where demographic, epidemiologic, economic, and political factors introduce significant uncertainty [12, 14].

Scenario‐based and simulation approaches are increasingly recommended for addressing such uncertainties, allowing planners to model the impacts of variables like demographic shifts or technological advancements on workloads and workflows [12]. The gradual introduction of the Workload Indicators of Staffing Need (WISN) modelling method, which allows skill‐mix planning, represents an important step forward. Although WISN has been implemented in various contexts, its success depends on reliable data, small‐scale pilot projects, and adherence to detailed guidelines [15, 18, 52]. These methods have been successfully used in other countries to anticipate workforce needs under changing conditions [9, 18, 53]. Countries with similar social and political contexts, including Armenia, Moldova and Ukraine, have explored strengthening health workforce planning with international partners, such as the World Health Organisation, through application of evidence‐based planning approaches [7, 54, 55].

While similar efforts could benefit Georgia, planning must account for the expected implementation challenges, many of which are specific to this country. Georgia's unique healthcare landscape differs significantly from that of other Easter European and low‐ and middle‐income countries. Georgia's healthcare system is highly privatised, with over 80% of hospital beds controlled by for‐profit entities, apart from a few exceptions such as rural ambulatories, psychiatric hospitals, and infectious disease care facilities [56]. Waves of deregulation, decentralisation and privatisation have led to a situation in which healthcare is provided by a high number of organisations with diverse internal policies and regulations, and a fragmented health information infrastructure. This represents a substantial challenge to data standardisation and management, and to policy implementation. While Georgia's governments have been working towards integrated health information technology systems, many challenges remain [56].

Hospitals in Georgia are often owned by entities that also control insurance and pharmaceutical businesses, creating conflicts of interest and challenges in data flow. Despite a relatively high number of physicians, the system struggles with meaningful reforms in nursing education and professional recognition, while the proliferation of medical schools and MD students has continued to be driven by for‐profit motives. As a post‐Soviet nation, Georgia inherited a hospital‐oriented centralised Soviet health system. Leading to an oversupply of hospitals and beds, which were later privatised in a chaotic transition. Limited experience in professional self‐governance, frequent political instability, and delays in reintroducing continuing medical education (CME) further complicate progress making Georgia's healthcare workforce planning uniquely complex.

In this context, implementation of hybrid planning methods (combining different methods tailored to local and organizational contexts) should also be considered. Given that modelling accuracy is influenced by data availability, quality, and planning duration [12], Georgia could adopt a dual strategy, focussing on mid‐term (2–3 years) planning while investing in long‐term solutions, such as establishing a national health workforce accounts system [57, 58]. Implementation of WISN could be rolled out starting with small‐scale pilot projects. More broadly, workforce planning should be viewed as a continuous process, where multiple frameworks and models evolve over time to address emerging challenges and opportunities.

Dentists, pharmacists, laboratory staff, epidemiologists, traditional and complementary medicine professionals and representatives of allied health professions, need to be included in the planning process in Georgia. This can be done for a single profession or speciality. However, on a broader scale, modelling should avoid singularity, and aim for integration in planning, service delivery (e.g., multidisciplinary and integrative care), and funding [12].

Beyond the technical aspects of health workforce planning, it is essential to recognize it as a political process [13]. Decisions within Georgia's health and education systems, along with other foundational sectors, are often influenced by political interests, which are in turn shaped by financial, business, economic, social, and cultural factors. These priorities and interests can vary significantly among political actors and between different government structures and ministries. The frequent turnover in political leadership and the policy changes driven by various influences can also impact health systems development and, consequently, health workforce planning. Beyond the health and education systems, which are critical in driving workforce demand and production, broader policies related to social and economic development, security, and migration must also be considered. The interests of various groups within the broader political and societal context are equally important. This underscores the need for an intersectoral, holistic approach to health workforce planning and strategy implementation [59]. Such efforts should be informed by research‐based evidence, and qualitative research is particularly valuable for navigating the complexities of political and policy influences.

Designing and implementing strategic health workforce plans in Georgia should expect individual, organisational, national and international implementation barriers requiring specific mitigation measures. We summarise some of the implementation barriers in the Supporting Information S1: Table 2.

Health workforce planning varies among the European countries, thus challenging cross‐system data utilization and broader European planning [19]. Furthermore, many countries experience challenges of insufficient data quality or availability, somewhat similar to the situation in Georgia. Health workforce planning initiatives in Georgia should be designed and implemented in close communication and collaboration with the country's strategic partners, such as the European Union, from early stages. This would allow the exchange of expertise and experience and align the country's objectives to that of the bigger EU family, contributing to smooth Georgia‐EU health policy harmonization, and alignment of local policies and plans to regional health workforce planning objectives and strategies.

## 5 Conclusion

The adoption and implementation of an effective, operational, appropriate and costed health workforce strategy in Georgia requires immediate investment in health workforce data collection and research to inform and rationalise health workforce planning and decision‐making, ensuring that workforce planning strategies are tailored to the local realities and circumstances.

Strengthening quality assurance in health services and personnel training needs to ensure that a competent and resilient workforce is capable of delivering high‐quality healthcare that meets both current and future population needs. A centralised unit dedicated to health workforce planning should lead, coordinate, and evaluate the implementation of an intersectoral and multi‐stakeholder agenda in the context of political, epidemiological, demographic, and economic dynamics. Collaboration with international partners, such as the EU and the WHO is recommended to exchange experience and align the local policies and plans to the regional health workforce planning objectives and strategies.

## Ethics Statement

The authors have nothing to report.

## Conflicts of Interest

The authors declare no conflicts of interest.

## Supporting information

Supporting Information S1

## Data Availability

Data sharing is not applicable to this article as no new data were created or analysed in this study.
