# Supplementary material for: Strengthening Health Workforce in Georgia: Identifying Gaps and Integrating Evidence‐Based Strategic Planning
Source: Int J Health Plann Manage. 2025 Mar 30;40(4):993–1001. doi: 10.1002/hpm.3922 (PMC12215597; doi:10.1002/hpm.3922)
Supplement: Supplementary file 1 — Supporting Information S1 [file HPM-40-993-s001.docx]

| **Planning Model** | **Description** | **Suitability** | **Example** | **Challenge** |
| --- | --- | --- | --- | --- |
| **Demand-Based Approach** | Focus on forecasting workforce needs based on healthcare demand patterns, influenced by market dynamics, patient preferences, and private sector growth. | Aligns with the market-driven nature of privatized systems, where patient demand and service profitability often dictate workforce requirements. | The use of market analyses and health service utilization trends to determine staffing needs. | May prioritize profitable services over equitable access. |
| **Supply-Demand Alignment** | These models assess both the supply of health professionals (e.g., graduates, migration trends) and the demand for their services. | Useful in systems where workforce supply is fragmented across private and public sectors. | Workforce projections that integrate private sector employment trends with public  health needs. | Requires robust data sharing between public and private entities. |
| **Needs-Based Approaches** | Focus on population health needs while incorporating private sector dynamics  to ensure alignment with national health goals. | Helps balance equity and efficiency by integrating health outcomes into  workforce planning. | Blended approaches where private sector incentives are aligned with public health  priorities. | Requires strong regulatory frameworks to guide private sector participation. |
| **Simulation and Scenario Planning** | Use simulations to model different scenarios based on privatization trends,  policy changes, and market forces. | Allows stakeholders to visualize the impact of various workforce policies and market developments. | Modeling the impact of introducing new private healthcare facilities on workforce distribution. | Data-intensive and dependent on accurate assumptions. |
| **Benchmarking** | Compare workforce planning strategies, outcomes, and policies across different regions, countries, or institutions to identify best practices and performance gaps. | Useful for continuous improvement in workforce planning by leveraging international or inter-organizational comparisons to refine strategies. | Comparing physician-to-population ratios, workforce retention rates, and task-shifting policies across countries with similar healthcare systems. | Differences in healthcare structures, funding models, and policy environments may limit direct applicability of benchmarking insights. |
| **Hybrid Methods** | Combines elements of demand-based, supply-based, and needs-based  approaches tailored to privatized systems. | Offers flexibility to adapt to diverse stakeholders and market-driven contexts. | Using supply-demand alignment models for urban areas and needs-based planning  for underserved regions. | Complexity in implementation and data integration. |

Supplement to the manuscript “Strengthening Health Workforce in Georgia’s Health System: Identifying Gaps and Integrating Evidence-Based Strategic Planning”

Table 1: Overview of the common health workforce planning approaches/models.

Table 2: Overview of the implementation **barriers** and **strategies** for overcoming them in the health workforce planning process in Georgia.

| **Implementation Barrier** | **Proposed Strategy** |
| --- | --- |
| **Data Challenges** | - Operate a national health workforce registry to track professionals. |
|  | - Standardize data collection methods and integrate private sector data. |
|  | - Work with WHO and international bodies to establish data standards. |
| **Political and Institutional Barriers** | - Develop long-term policy frameworks that ensure continuity across political cycles. |
|  | - Embed health workforce planning into national development strategies to minimize political influence. |
| **Private Sector Resistance** | - Create a public-private partnership framework with incentives for private entities to engage in workforce planning. |
|  | - Encourage data sharing and collaboration through financial or regulatory incentives. |
| **Training and Education Infrastructure Constraints** | - Expand residency programs, mentorship, and CME opportunities to meet healthcare needs. |
|  | - Invest in medical school infrastructure and bedside training opportunities. |
|  | - Develop, introduce, and implement robust and objective quality assurance mechanisms for undergraduate, postgraduate and continuing medical education |
| **Fragmented Health Information System** | - Develop an integrated health information system for public and private sectors to share workforce data. |
|  | - Prioritize the creation of interoperable systems that ensure seamless data flow. |
| **Political Instability** | - Embed workforce planning into laws and regulations to safeguard against frequent political priority changes. |
|  | - Focus on building long-term commitments from all political actors involved. |
| **Uncertainty in Workforce Demand** | - Implement scenario-based forecasting and simulation modeling to account for uncertainty. |
|  | - Use tools like WISN for small-scale testing and refinement before scaling nationwide. |
| **Pilot Project Feasibility** | - Launch small-scale pilot projects to test new workforce strategies in specific regions or specialties. |
|  | - Collect feedback and refine the model based on pilot outcomes before full-scale implementation. |
| **Monitoring and Evaluation (M&E)** | - Establish clear performance indicators to track progress on workforce goals. |
|  | - Regularly assess and adjust the strategy based on the evaluation outcomes. |

|  |  |
| --- | --- |
|  |  |
